# Ancillary benefits of antenatal ultrasound: an association between the introduction of a low-cost ultrasound program and an increase in the numbers of women receiving recommended antenatal treatments

**DOI:** 10.1186/s12884-014-0424-9

**Published:** 2014-12-19

**Authors:** Andrew B Ross, Kristen K DeStigter, Anastasia Coutinho, Sonia Souza, Anthony Mwatha, Alphonsus Matovu, Michael Grace Kawooya, Ssembatya Renny

**Affiliations:** Department of Radiology, University of Vermont Medical Center, 111 Colchester Rd., Burlington, Vermont 05401 USA; University of Vermont College of Medicine, 89 Beaumont Ave., Burlington, Vermont 05405 USA; Clinical Research Division, Philips Healthcare, 22100 Bothell Everett Hwy, Bothell, Washington 98021 USA; Department of Surgery, Mubende Regional Referral Hospital, Mubende, Uganda; Ernest Cook Ultrasound Research and Education Institute, Mengo Hospital, Sir Albert Cook Rd., Kampala, Uganda; Imaging the World Africa, Nayla-Namugongo Rd., Nayla, Uganda

**Keywords:** Maternal health, Neonatal health, Antenatal care, Global health, Antenatal ultrasound

## Abstract

**Background:**

In June of 2010, an antenatal ultrasound program was introduced to perform basic screening examinations at a health care clinic in rural Uganda. The impact of the program on the existing antenatal care infrastructure including the proportion and number of women receiving recommended antenatal care at clinic visits was unknown. The aim of this study was to investigate the relationship between the advent of the ultrasound program and the proportion of women receiving recommended antenatal interventions at their clinic visits. Change in the absolute numbers of antenatal services provided was also assessed.

**Methods:**

Records at the Nawanyago clinic were reviewed to determine the total numbers of women receiving specific interventions before and after the advent of the ultrasound program including HIV testing, intermittent preventive therapy for malaria, presumptive anti-parasitic treatment, and provision of iron and folate for anemia. The rate at which these interventions were provided (number of interventions per clinic visit) was also assessed. The differences in absolute numbers of antenatal interventions before and after the introduction of the ultrasound program were assessed using the Wilcoxon rank-sum test. Differences in intervention rate were assessed using negative binomial regression modeling.

**Results:**

The mean monthly numbers of women receiving each of these interventions increased significantly with the greatest increase seen in numbers of women receiving anemia and deworming treatments at +113% and +102% respectively (p < 0.001). The intervention rate increased for anemia treatment, deworming treatment, and 2nd dose of intermittent preventive therapy for malaria. A slight decrease in intervention rate was observed for 1st dose of malaria treatment with a rate ratio of 0.88 (0.79 - 0.98, 95% CI). Intervention rate for HIV testing was not significantly changed.

**Conclusion:**

The introduction of a low-cost antenatal ultrasound program at a health care clinic in rural Uganda was associated with increases in the number of women receiving specific recommended antenatal care interventions. Effect on intervention rates was mixed but showed an overall increase. The use of ultrasound in this context may provide a benefit to the maternal and neonatal health of the community.

## Background

Despite concerted effort from the global public health community, the developing world continues to bear a disproportionate burden of maternal and neonatal morbidity and mortality. In sub-Saharan Africa, women face a 1 in 39 lifetime risk of dying during childbirth. In the developed world, the risk is 1 in 3,800 [1]. Like other countries in the region, Uganda has seen some improvement in maternal mortality ratio (MMR, defined as the number of maternal deaths per 100,000 live births) since 1990, the year in which the Millennium Development Goals were adopted by the United Nations [2]. Between 1990 and 2010 MMR had declined from 600 to 310 deaths per 100,000 live births [3]. Despite this improvement, MMR remains unacceptably high and falls short of the 75% improvement mandated by MDG 5. Neonatal and childhood mortality throughout the region likewise remains high with 99 deaths per 1000 live births in Uganda in 2010. Although this represents a reduction in childhood deaths since 1990, in recent years an increasing proportion of these deaths are occurring in children in the first few months of life indicating a need for risk reduction in the neonatal time period [1]. Concern has been expressed over whether the Millennium Development Goals can be achieved by the 2015 target date [4,5].

Effective interventions for achieving further reduction in MMR and neonatal mortality have been identified and include a focus on increasing access to skilled antenatal care and assistance at delivery [6,7]. A skilled birth attendant (SBA) working within an effective health care system can frequently manage many of the most common causes of maternal mortality including hemorrhage, sepsis, and obstructed labor. Countries that have increased the numbers of births attended by SBAs have seen an associated decrease in MMR [8]. Antenatal clinic (ANC) visits also provide an opportunity for the implementation of clinical services and education and may have a positive impact on encouraging women to return to the clinic for skilled care at delivery [9].

The need for skilled antenatal care has been consistently emphasized as a strategy for reduction of maternal and neonatal mortality, but increasing access has been difficult [10]. Although Uganda has seen an overall increase in health care resources in the last decade—in 2010, 72% of the population lived within 5 km of a health care facility compared with 49% in 2000—access to skilled care remains split with the poor and those living in rural areas significantly less likely to have access to health care resources [11]. Efforts to provide skilled maternal care to women in rural areas have faced challenges. Women may have limited transportation and payment options and may be influence by traditional attitudes regarding pregnancy [12]. The need for strategies to encourage women to come to clinics for skilled care during pregnancy and at delivery remains acute.

The Uganda Ministry of Health recommends four ANC visits based on the WHO model [13]. Recommended interventions include blood pressure monitoring, acute illness treatment, tetanus vaccination, screening for syphilis, counseling and screening for HIV, prevention of mother-to-child transmission of HIV in seropositive patients, presumptive treatment for parasites, screening for anemia and provision of iron and folate supplementation, and adherence to a two dose schedule of intermittent preventative treatment (IPT) for malaria. Additionally women can be counseled regarding safe birthing and infant care practices, and other resources, such as insecticide-treated bed nets can be distributed if available. These interventions have the potential to improve birth outcomes in both mothers and their children and represent opportunities to make progress towards MDG 4 and 5. However, the quality of ANC visits is a concern and some reviewers have concluded that despite improving coverage rates, ANC visits may not provide recommended interventions and may fail to prevent, diagnose, or treat complications [14,15]. A survey in Uganda showed inconsistent implementation of ANC care. Although some interventions such as blood pressure monitoring were widely performed, fewer than half of respondents had been offered HIV testing and only two thirds had received IPT for malaria [16]. There remains a clear need for strategies both to increase the numbers of women receiving skilled antenatal care and to increase the rate at which recommended interventions are provided at the time of these ANC visits.

### The role of ultrasound in pregnancy

The role of antenatal ultrasound in the developing world has been controversial with several investigators concluding that it provides only a modest benefit not felt to be worth the cost of the programs [17,18]. However, these investigations utilized Western style ultrasound programs with the use of full-sized ultrasound machines operated by trained sonographers and image interpretation provided by skilled radiologists or obstetricians. Additionally they were not powered to examine maternal and neonatal mortality, and whether antenatal ultrasound can impact these clinical outcomes in the developing world remains an open question. Consequently there has been a call to evaluate the use of more affordable, sustainable methods of providing antenatal ultrasound in this environment [19].

In our previous research, we described the logistics of implementing a low-cost, self-sustaining antenatal ultrasound program in low resource environments [20]. In June of 2010, the NGO Imaging the World implemented this type of program at the Nawanyago community level III health care center (HC III) in Uganda. The introduction of a new technology into this type of environment must be done cautiously, and our initial investigation was to examine the impact of the ultrasound program on the numbers of women coming to the clinic for antenatal care and skilled care at delivery. In that work, we demonstrated an apparent “magnet effect” of ultrasound with a significant increase in number of women coming to the clinic for antenatal care and delivery [21]. Although this increase in health care utilization is an important end point, the full impact of this new ultrasound program on existing antenatal programs merits further evaluation. The aim of our current study was to assess the impact of the ultrasound program on the numbers of women receiving specific antenatal interventions and to assess the rate at which these interventions were provided at ANC visits. Given that the numbers of women coming to the clinic increased significantly following the introduction of the ultrasound program, it would be expected that the absolute numbers of interventions provided would also increase. Quantifying these interventions allows for a tangible measure of an indirect result of introducing this new technology. Assessment of the rate at which women coming to the clinic receive recommended interventions is a more important endpoint and a marker of ANC visit quality. It is important to demonstrate that the use of antenatal ultrasound does not detract from provision of existing effective care.

## Methods

The logistics of the ultrasound program in use at Nawanyago HC III have been well described in our previous work [20,21]. In brief, the program uses low-cost, portable ultrasound machines that can easily be transported and repaired. Scans are offered at the time of the first ANC visit and again at 32 weeks gestation or based on clinical presentation. To address the human resource problem of few trained sonographers in this low resource setting, scanning protocols have been developed that rely solely on surface anatomy landmarks. The ultrasound probe is passed over the gravid abdomen in a series of six prescribed sweeps and records a series of volumetric images that can be scrolled through by the reviewer like a short video. Earlier research has demonstrated the images to be of diagnostic quality [22]. The images are compressed and sent via cell phone modem to a remote Internet server where they can be accessed by a credentialed reviewer for interpretation. An abbreviated report is sent to the nurse midwife via SMS text messaging with the full report to follow by email. Patients are able to receive their exam results prior to leaving the clinic. A small fee (approximately $2 USD) is charged for the ultrasound scan, which allows the program to be self-sustaining. The price was determined based on local community standards. Fetal gender is not disclosed.

Ultrasound can reliably identify many of the most common causes of neonatal and maternal morbidity and mortality including placenta previa, multiple gestations, and causes of obstructed labor [23]. Early identification of high-risk conditions of pregnancy allows providers to recommend delivery at the clinic under the supervision of a skilled midwife or make a referral to a higher level of care. Women identified as needing urgent or emergent care beyond what can be provided at the clinic, including C-section, are referred to Kamuli Mission Hospital, a distance of 24 km away.

### Data collection

Available data consisted of aggregated monthly counts of anemia, deworming, and IPT1 and IPT2 treatments, and HIV testing from January 2007 through April 2012. Data on anemia and deworming treatment were available for the 34 months preceding the ultrasound (i.e. from August 2007 through May 2010); data on HIV testing were available for 24 months prior to the ultrasound installation (June 2008-May 2010) data for IPT1 were available for 40 months and data for IPT2 were available for 36 months prior to ultrasound. The data collection period following the introduction of the ultrasound program was 22 months. Data for other interventions such as tetanus vaccination, blood pressure monitoring, etc. were not available. The irregular availability of data is not ideal but reflects the realities of research in a low resource environment. Clinic staff provided data to the researchers, and the clinic records were independently reviewed by a research associate and found to be concordant to the provided data.

### Statistical analysis

The endpoints of this study were twofold. The first endpoint was the absolute numbers of women receiving these specific antenatal interventions expressed as mean numbers of interventions per month. The second endpoint was the intervention rate defined as the total numbers of each intervention provided per antenatal visit. The relationship between number of monthly ANCs and this ultrasound program was the subject of a previous publication [21].

Prior to statistical analysis all data were assessed for normality by visual inspection of normal probability plots and formally by the Shapiro-Wilk test. In all but two cases (number of deworming treatments and number of IPT1 treatments), data were not normal. Therefore, Wilcoxon rank-sum tests were used to test the null hypothesis of no differences in the distribution of each antenatal intervention prior to and after the ultrasound program.

To determine whether there was a difference in the rate of intervention delivery, Poisson regression modeling for each intervention was employed. The number of interventions was modeled as the response variable and the presence/absence of ultrasound as the predictor of interest with the total number of ANC visits included as a covariate. Models were assessed for fit and dispersion of the variance parameter, in all cases, the variance parameter was over dispersed, hence Negative Binomial regression models were fit instead. Model fit was assessed using the ratio of deviance to degrees to freedom and inspection of residuals plots. All statistical analyses were performed using SAS 9.3 software (SAS Institute Cary, NC).

### Ethics statement

This study was approved by the local institutional review board (IRB) at Mengo Hospital (Protocol title: Evaluation of Simple Ultrasound Protocols for Improving Access to Ultrasound in Low Resource Settings, study number 013/05-10). All women who received an ultrasound scan provided written informed consent to have their data included in the research cohort. Appropriate translation and literacy services were provided when needed. Data from clinic patients prior to the start of the ultrasound program was included in the study as an historic control group and was used in aggregate fashion only. The consent form and process and the use of historic control data was approved by the IRB. All ultrasound images and patient records were de-identified with use of a medical record number system. Personally identifiable patient records were kept in a secure location at Nawanyago HC III. All data were coded anonymously in aggregate fashion for analysis. Women were provided their ultrasound examination results in written form in the Uganda Ministry of Health Maternal Passport, which is distributed by the HC III to all pregnant patients. For ethical reasons, fetal gender is not disclosed and the program is periodically audited to ensure compliance with this requirement.

## Results

**Figure 1:**
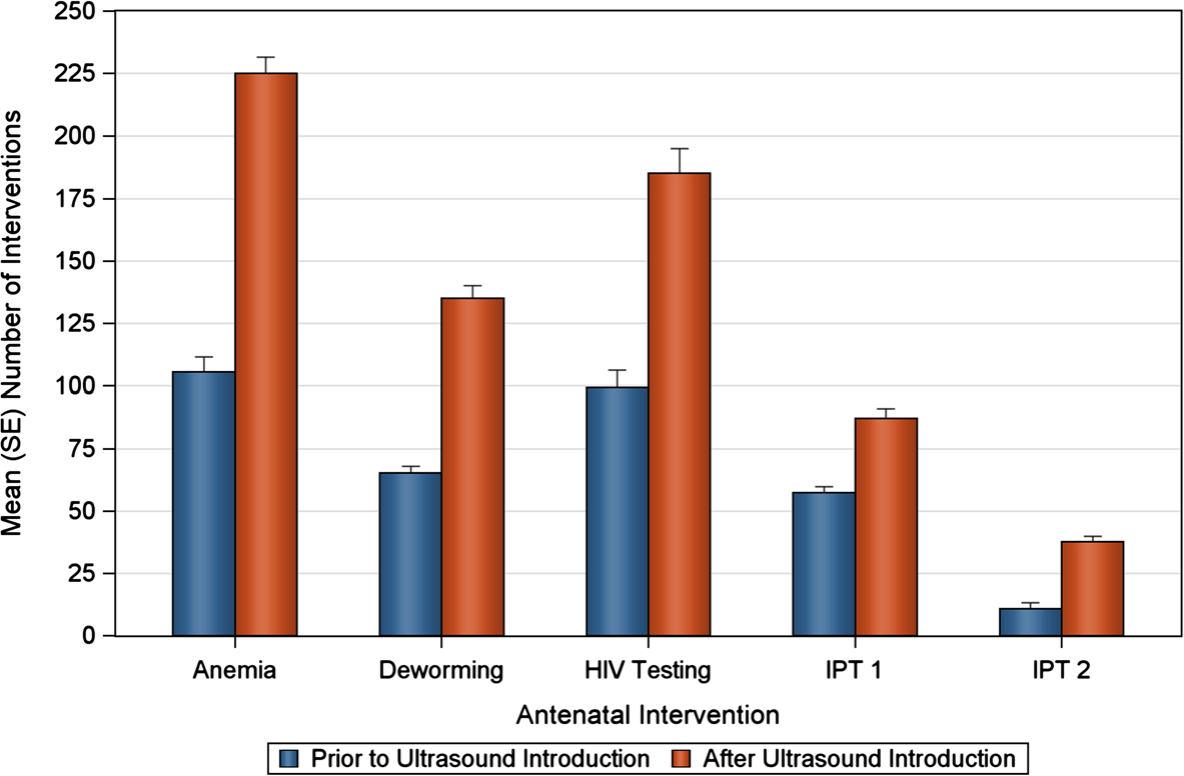
**Mean monthly numbers of women receiving specific antenatal interventions at the Nawanyago HC III are shown before and after the introduction of ultrasound.** Statistically significant increases occurred following the introduction of the ultrasound screening program in the mean number of women receiving anemia treatment, deworming treatment, HIV testing, and IPT 1 and 2.

**Figure 2:**
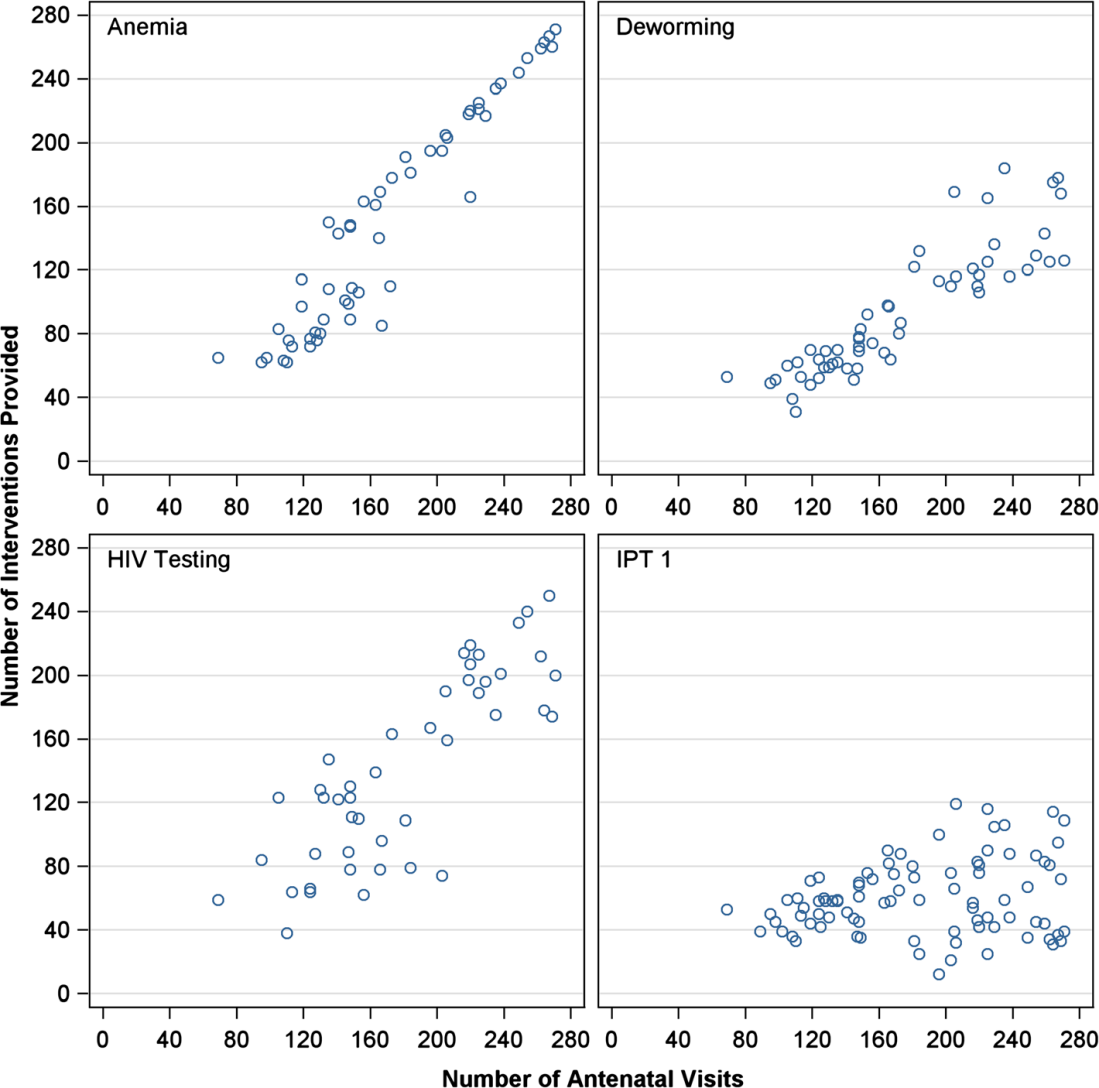
**The number of antenatal interventions provided is plotted against the number of ANC visits.** Increase in number of ANC visits was correlated with number of antenatal interventions provided, both pre and post ultrasound, this correlation was strongest for anemia treatment, HIV testing and deworming treatment.

Table 1 summarizes the number of interventions provided prior to and after the ultrasound program. For all interventions, significant increases in the total number were observed. Both the mean and median number of anemia, deworming and IPT2 treatments more than doubled. Increases in the number of HIV tests conducted were also notable; median number increased from 110 to 197 (80%). IPT1 treatments increased modestly relative to the other interventions, a 43% increase in the median value was observed. Figure 1 shows the total number of interventions provided. All the years subsequent to the ultrasound program have more interventions provided. Figure 2 shows the number of interventions provided plotted against the number of ANC visits. Increase in number of ANC visits was correlated with number of antenatal interventions provided, both pre and post ultrasound, this correlation was strongest for anemia treatment, HIV testing and deworming treatment.

**Table 1:** **Number of monthly interventions at Nawanyago health center pre and post ultrasound program**

**Antenatal intervention**	**Period**	**Number months**	**Mean (SD)**	**Median**	**Min max**	**95% CI**	**p-value**
Anemia	Pre Ultrasound	34	105.5 (35.8)	98.0	62 - 178	93 - 118	<.0001
	Post Ultrasound	22	225 (30.4)	221.0	166 - 271	211.2 - 238.8	
Deworming	Pre Ultrasound	34	65.2 (15.4)	63.0	31 - 98	59.8 - 70.6	<.0001
	Post Ultrasound	23	135 (24.9)	125.0	106 - 184	124.3 - 145.8	
HIV testing	Pre Ultrasound	24	99.3 (33)	96.0	38 - 163	85.1 - 113.6	<.0001
	Post Ultrasound	23	185.3 (46.3)	196.5	74 - 250	164.8 - 205.8	
IPT1	Pre Ultrasound	40	57.4 (14.7)	58.0	33 - 90	52.7 - 62	<.0001
	Post Ultrasound	23	87.1 (18.2)	83.0	57 - 119	79.2 - 95	
IPT2	Pre Ultrasound	36	10.9 (14.3)	7.0	0 - 72	6.2 - 15.7	<.0001
	Post Ultrasound	23	37.7 (10.8)	39.0	12 - 59	33 - 42.3	

P-values are based on two-sided Wilcoxon Rank Sum Tests.

**Figure 3:**
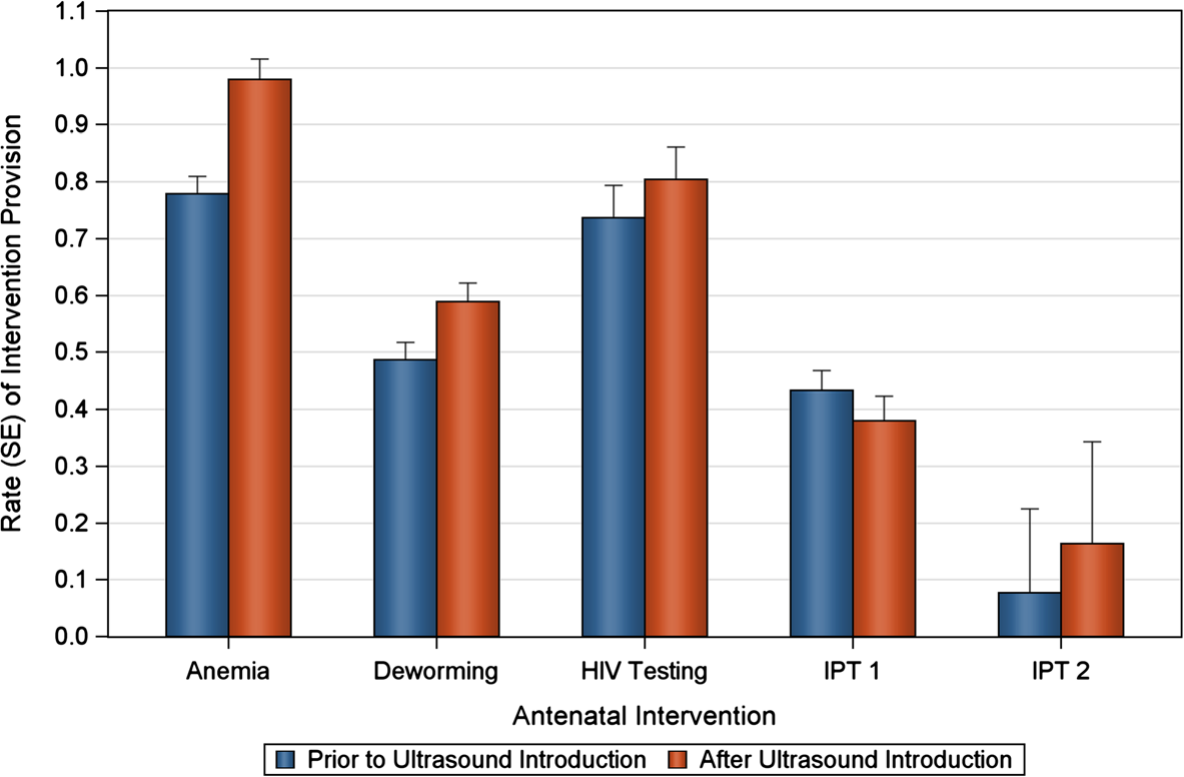
**The rate of antenatal intervention (number of antenatal treatments per antenatal clinic visit) is shown before and after the introduction of the ultrasound program.** Significant increases were seen in the proportion of women receiving anemia treatment, deworming treatment, and IPT 2. A slight but statistically significant decline was observed in the rate of IPT 1 administration. HIV testing was increased but not to the level of statistical significance.

Increases in the rates of intervention provision were observed for all interventions except IPT1, which declined from 0.45 treatments per visit to 0.38 (Table 2 and Figure 3). For anemia the rates ranged from 0.5 to 1.1 treatments per visit, while HIV testing ranged from 0.4 to 1.2, values greater than 1 suggest that women may have brought family members for their ANC visits or were being tested repeatedly during some months.

**Table 2:** **Changes in antenatal intervention rate at Nawanyago health center pre and post ultrasound program**

**Antenatal intervention**	**Period**	**Number months**	**Rate**	**Standard error**	**95% CI**	**Rate ratio**	**P-value**
Anemia	Pre Ultrasound	34	0.78	0.030	0.73 - 0.83	1.26 (1.15 - 1.38)	<.0001
	Post Ultrasound	22	0.98	0.034	0.92 - 1.05		
Deworming	Pre Ultrasound	34	0.49	0.031	0.46 - 0.52	1.21 (1.11 - 1.32)	<.0001
	Post Ultrasound	23	0.59	0.033	0.55 - 0.63		
HIV Testing	Pre Ultrasound	24	0.80	0.060	0.71 - 0.90	1.09 (0.93 - 1.28)	0.2742
	Post Ultrasound	23	0.81	0.060	0.72 - 0.91		
IPT1	Pre Ultrasound	40	0.45	0.039	0.42 - 0.49	0.88 (0.79 - 0.98)	0.0186
	Post Ultrasound	23	0.38	0.048	0.35 - 0.42		
IPT2	Pre Ultrasound	36	0.09	0.161	0.07 - 0.12	2.13 (1.35 - 3.35)	0.0012
	Post Ultrasound	23	0.16	0.196	0.11 - 0.24

The models estimating relative rate ratios comparing ultrasound to no ultrasound were significant for anemia, RR = 1.26 (1.15 – 1.38); deworming, RR = 1.21 (1.11 – 1.32); and IPT2, RR = 2.13 (1.35 – 3.35). The relative rate ratio for HIV testing was not significantly greater than 1 and a significant decrease in the relative rate ratio for IPT1 was also observed (RR = 0.88, 0.79 – 0.98).

## Discussion

In our earlier work we demonstrated an apparent “magnet effect” following the introduction of an antenatal ultrasound program in a low-resource, ultrasound naïve area with more women coming to ANC visits and deliveries. This increase in the number of ANC visits also represents a large increase in the numbers of women receiving recommended antenatal care including HIV testing and treatment, deworming, anemia prophylaxis, and IPT for malaria that we were able to quantify in this study. Given the benefit these interventions have been shown to have in prior studies, this represents a significant impact on the maternal and neonatal health of this community.

This study addresses an important additional concern regarding the impact of an ultrasound program on quality of antenatal care in an ultrasound naïve community. That is, could the presence of ultrasound or the increased numbers of women coming to the clinic for ANC visits dilute the quality of antenatal care provided. This study shows that the introduction of ultrasound did not “crowd out” other antenatal interventions. In fact, ultrasound was associated with an almost across the board increase in the proportion of women receiving recommended interventions at their ANC visits. This unexpected increase in ANC visit quality may have resulted from increased attention paid to ANC visits associated with the training and preparation for the use of ultrasound.

It is important to note that this study replicated findings from other studies showing that not all recommended antenatal care is provided at any given ANC visit. In the post ultrasound period while prophylactic supplementation for anemia was provided at 98% of visits only 16% of visits provided IPT2 treatment for malaria. To some extent this reflects that supplementation for anemia is recommended for every ANC visit whereas both IPT1 and IPT2 are administered at one of the four ANC visits over the course of the pregnancy. Thus overall the proportion of women receiving a complete course of IPT1 and IPT2 would be higher than is reflected in our study. The reasons for the slight decline observed in proportion of visits at which IPT1 was provided are unclear and must be investigated further. This conflicts with the rest of the data that show a stable or increasing proportion of visits at which interventions were provided.

Our study has weaknesses that should be acknowledged. This study utilizes an historic control group and thus any conclusions of causality should be tempered with caution. As best as could be determined by the investigators and in consult with clinic staff, conditions at the clinic and in the surrounding community including political climate, population, weather trends, and infrastructure were stable over the study period. Specifically, the population of Nawanyago sub-county was relatively unchanged: 21,038 in the 2002 census increasing only to 24,058 in the 2014 census [11]. Nonetheless the possibility of confounding cannot be entirely eliminated with a study of this design. Additionally, if we are to conclude that this study represents a true increase in the number of women receiving antenatal care, we must assume that they would not have otherwise received care at another health care center. Given the lack of other facilities within easy reach of this region, this seems like a reasonable assumption. However, in further studies we plan to more closely assess the health care seeking behaviors of pregnant women at the clinic to understand the role ultrasound played in increasing attendance at ANC visits.

Despite these weaknesses, the increase in antenatal interventions is robust, well timed with the advent of the ultrasound program, and durable throughout the follow up period. Additionally, the overall increase in quality of ANC visit following the introduction of the ultrasound program is reassuring that the availability of ultrasound at antenatal visits did not negatively impact other antenatal interventions and may in fact have been a driver for increased quality of care.

## Conclusion

In the long term it will be important to assess the direct impact of antenatal ultrasound on clinical outcomes for pregnant women and their children, and indeed this is the focus of a longitudinal outcomes study at the Nawanyago site. However, the introduction of technology into a low resource environment must be done cautiously and other factors such as the impact on the existing health care framework must be considered. This study builds on our previous work demonstrating that the introduction of an antenatal ultrasound program was associated not only with an increase in the numbers of women being provided antenatal care but also with an increase in the quality of the antenatal care being provided. A well-integrated ultrasound program working within an effective health care system has the potential to significantly impact the health of the surrounding community.

## Abbreviations

ANC: Antenatal clinic
HC III: Level three health care center
HIV: Human immunodeficiency virus
IPT: Intermittent preventive therapy for malaria
MDG: Millennium development goals
MMR: Maternal mortality ratio
NGO: Non-governmental organization
SBA: Skilled birth attendant
SMS: Short message service
USD: United States dollar
WHO: World Health Organization
